# Prognostic effect of immunohistochemically determined molecular subtypes in gastric cancer

**DOI:** 10.1186/s12885-024-13236-z

**Published:** 2024-12-02

**Authors:** Jefim Brodkin, Tuomas Kaprio, Jaana Hagström, Alli Leppä, Arto Kokkola, Caj Haglund, Camilla Böckelman

**Affiliations:** 1 Translational Cancer Medicine Research Program, Faculty of Medicine, University of Helsinki, PO Box 340, Haartmaninkatu 4, Helsinki, HUS FIN-00029 Finland; 2 Department of Surgery, University of Helsinki and Helsinki University Hospital, Helsinki, Finland; 3 Department of Pathology, University of Helsinki and Helsinki University Hospital, Helsinki, Finland; 4 Department of Oral Pathology and Radiology, University of Turku, Turku, Finland

**Keywords:** Gastric cancer, Immunohistochemistry, Survival, Epstein–Barr virus

## Abstract

**Introduction:**

Gastric cancer is the fifth most common cancer worldwide and the fourth most common cause of cancer-related death. Two molecular subtyping classifications were recently introduced: The Cancer Genome Atlas (TCGA) and the Asian Cancer Research Group (ACRG) classifications.

**Methods:**

We classified a cohort of 283 gastric cancer patients undergoing surgery at Helsinki University Hospital between 2000 and 2009. We constructed a tumour tissue microarray immunostained for the following markers: microsatellite instability (MSI) markers MSH2, MSH6, MLH1, and PMS2; p53; E-cadherin; and EBER*ISH*.

**Results:**

In the univariate survival analysis for disease-specific survival, the Epstein–Barr virus (EBV) -positive subtype exhibited the worst prognosis with a hazard ratio (HR) of 2.49 (95% confidence interval [CI] 1.19–5.25, *p* = 0.016) compared with the most benign subtype, chromosomal instability (CIN). Using TCGA’s classification, the genetically stable (GS) and MSI subtypes exhibited a worse survival compared with CIN (HR 1.73 [95% CI 1.15–2.60], *p* = 0.009 and HR 1.74 [95% CI 1.06–2.84], *p* = 0.027, respectively). Using the ACRG classification, the p53 aberrant subtype exhibited the best prognosis, whereas wild-type p53, MSI, and the epithelial–mesenchymal transition (EMT) subtypes exhibited poorer prognoses (EMT: HR 1.90 [95% CI 1.30–2.77], *p* < 0.001) when compared with aberrant p53.

**Conclusions:**

Immunohistochemical analysis can identify prognostically different molecular subtypes of gastric cancer. The method is inexpensive and fast, yet reveals significant information for clinical decision-making. However, our study did not find that either molecular subtyping performed better than the other classification. Thus, further development of the most optimal grouping of different molecular subtypes is still needed.

## Introduction

Gastric cancer (GC) is a common cancer worldwide. While its incidence is decreasing in the Western world, it remains one of the most common forms of cancer. Gastric cancer has the fifth highest incidence globally and is the fourth most common cause of cancer-related death in the world [1].

The decreasing incidence of gastric cancer in the Western world in recent decades primarily results from the decrease in the prevalence of at least two known risk factors, *Helicobacter pylori* infection and smoking [2, 3]. That said, the prognosis of gastric cancer has remained poor: in 2017–2019 five-year survival in Finland barely exceeded 25% [4]. This low survival rate largely stems from late diagnosis and insufficient treatment options for advanced disease. New treatments and earlier diagnostics are needed to improve overall survival.

Gastric cancer is traditionally divided into intestinal, diffuse, and mixed subtypes according to the histological Laurén classification [5]. The World Health Organization offers a more specific classification, consisting of the papillary, tubular, mucinous, poorly cohesive, and uncommon histological types [6].

Recently, The Cancer Genome Atlas Research Network (TCGA) published a model of four molecular subtypes based on key genes dysregulated in each subtype [7]. The most common subtype, chromosomal instability (CIN), is identified by mutated *TP53* and mutations in different parts of the RTK–Ras signalling pathway, such as ERBB2, ERBB3, and KRAS. The microsatellite instability (MSI) subtype is identified by mutations in MSH2, MSH6, PMS2, or MLH1. The Epstein–Barr virus–associated (EBV) subtype is characterised by the *PIK3CA* mutation and *CDKN2A* silencing. The fourth subtype, genetically stable (GS), is associated with *CDH1* and *RHOA* mutations, both of which are associated with cell-to-cell junctions and cellular motility. Similarly, the Asian Cancer Research Group (ACRG) proposed another classification of molecular subtypes. The first subtype is also MSI-positive. The second subtype is the epithelial–mesenchymal transition (EMT), defined through E-cadherin staining and associated with downregulated cell proliferation. The third and fourth subtypes are p53-positive and p53-negative, respectively, found via p53 staining, fell into two categories: wild-type p53 and aberrant p53, respectively. Patients with an abnormal p53 expression were associated with other mutations in the *ERBB2* and *MYC* oncogene, for instance, whereas the p53-positive subtype associates with EBV positivity [8]. Gastric cancer is, like other cancers, genetically rather diverse, with an individual mutational burden significantly affecting the phenotype and disease progression in each individual patient.

Because methods for determining molecular subtypes using exome sequencing or other advanced methods are time-consuming, expensive, and difficult to interpret, it is important to identify easier ways to determine subtypes. Therefore, the use of immunohistochemical (IHC) biomarkers has been suggested as a way to identify surrogate markers to divide gastric cancer samples into molecular subtypes [9–12].

In this study, we aimed to find a set of marker proteins with which we could reliably subtype histological samples. This may reveal a novel way of classifying gastric cancer which is easier to translate into clinical decision-making compared with genome sequencing methods.

## Materials and methods

### Patients

The patient cohort comprised 283 individual patients operated on for histologically verified gastric adenocarcinoma in the Department of Surgery at Helsinki University Hospital between 2000 and 2009. We excluded patients with a history of malignant disease or synchronous cancer. The median age of the patient cohort at the time of surgery was 67.4 years (interquartile range [IQR] 56.9–76.8; Table 1). Among patients, 136 (49.1%) were male, and the median survival was 2.30 years (IQR 0.76–8.29). For staging, we used the seventh version of the tumour-node-metastasis (TNM) classification [13]. Adjuvant chemotherapy was administered to 110 patients (39.7%) and 45 patients (16.2%) received adjuvant radiotherapy. Only 10 patients (3.6%) received neoadjuvant chemotherapy.

**Table 1:** Associations between TCGA subtypes and clinicopathological variables

	**Total**	**CIN**	**GS**	**MSI**	**EBV**	*p* value^a^
	*n* = 277	*n* = 77 (27.8%)	*n* = 137 (49.5%)	*n* = 51 (18.4%)	*n* = 12 (4.3%)	
**Age**						
< 67	137	27 (19.7)	83 (60.6)	23 (16.8)	4 (2.9)	**0.002**
≥ 67	140	50 (35.7)	54 (38.6)	28 (20.0)	8 (5.7)	
**Sex**						
Male	136	45 (33.1)	56 (41.2)	26 (19.1)	9 (6.6)	**0.021**
Female	141	32 (22.7)	81 (57.4)	25 (17.7)	3 (2.1)	
**Stage**						
I	49	20 (40.8)	25 (51.0)	4 (8.2)	0 (0.0)	0.070
II	64	20 (31.3)	25 (39.1)	16 (25.0)	3 (4.7)	
III	105	24 (22.9)	55 (52.4)	19 (18.1)	7 (6.7)	
IV	58	13 (22.4)	32 (55.2)	12 (20.7)	1 (1.7)	
**Tumour invasion (pT)**						
1	37	15 (40.5)	21 (56.8)	1 (2.7)	0 (0.0)	0.050
2	41	16 (39.0)	15 (36.6)	8 (19.5)	2 (4.9)	
3	87	24 (27.6)	43 (49.4)	16 (18.4)	4 (4.6)	
4	112	22 (19.6)	58 (51.8)	26 (23.2)	6 (5.4)	
**Lymph node metastases (pN)**						
No	87	31 (35.6)	39 (44.8)	17 (19.5)	0 (0.0)	**0.045**
Yes	181	45 (24.9)	91 (50.3)	34 (18.8)	11 (6.1)	
**Distant metastases (M)**						
No	219	64 (29.2)	105 (47.9)	39 (17.8)	11 (5.0)	0.456
Yes	58	13 (22.4)	32 (55.2)	12 (20.7)	1 (1.7)	
**Laurén classification**						
Intestinal	111	77 (69.4)	0 (0.0)	24 (21.6)	10 (9.0)	N/A
Diffuse and mixed	166	0 (0.0)	137 (82.5)	27 (16.3)	2 (1.2)	
**E-cadherin expression**						
No staining	57	4 (7.0)	40 (70.2)	13 (22.8)	0 (0.0)	** < 0.001**
< 10%	31	3 (9.7)	16 (51.6)	8 (25.8)	4 (12.9)	
10–50%	58	13 (22.4)	29 (50.0)	12 (20.7)	4 (6.9)	
> 50%	128	55 (43.0)	51 (39.8)	18 (14.1)	4 (3.1)	
**Ki67 expression**						
< 10%	71	15 (21.1)	35 (49.3)	18 (25.4)	3 (4.2)	0.176
≥ 10%	156	53 (34.0)	67 (42.9)	27 (17.3)	9 (5.8)	
**p53 expression**						
Aberrant	213	63 (29.6)	101 (47.4)	41 (19.2)	8 (3.8)	0.372
Wild-type	62	13 (21.0)	35 (56.5)	10 (16.1)	4 (6.5)	

*Abbreviations*: *TCGA* The Cancer Genome Atlas, *CIN* chromosomal instability, *GS* genetically stable, *MSI* microsatellite instability, *EBV* Epstein–Barr virus-positive

^a^Pearson's chi-square

### Preparation of tissue samples

Tumour samples from patients were fixed in formalin, embedded in paraffin, and subsequently stored in the Department of Pathology at Helsinki University Hospital. Samples were collected from the archive and given an identification number to enable anonymous analysis. Tissue microarrays (TMAs) were constructed by taking 1-mm-diameter punches from the original paraffin blocks and embedding them into new blocks using an automatic TMA instrument (TMA Grand Master, 3D Histech Ltd.) [14].

### Immunohistochemistry

Paraffin TMA blocks were cut into 4-µm-thick sections, which were dried at 60 °C for 1 to 2 h before staining. Subsequently, the slides were deparaffinised with Sakura Tissue-Tek DRS. Antigen retrieval was performed in a PreTreatment module (Agilent Dako, CA, USA) with a pH 9 or pH 6 retrieval solution (Envision Flex target retrieval solution, DM828 or DM829, Agilent Dako), depending upon the primary antibody, for 15 min at 98 °C. Sections were blocked for 5 to 25 min (EnVision Flex peroxidase-blocking reagent, SM801). Paraffin sections were stained using an Autostainer 480S (LabVision Corp. Fremont, CA, USA), and slides were incubated at 37 °C for 40 min with primary antibodies (Additional files 1 and 2). Subsequently, all slides underwent a 30-min incubation period with a peroxidase-conjugated EnVision Flex/HRP (SM802) rabbit/mouse (ENV) reagent. Slides were visualised using DAB chromogen (EnVision Flex DAB, DM827) for 10 min. Mayer’s hematoxylin (S3309, Dako) was used for counterstaining. Mismatch repair (MMR) stainings MLH1, MSH2, MSH6, and PMS2 were stained in the routine HUSLAB diagnostic laboratory using an automatic Roche Ventana BenchMark ULTRA (F. Hoffman-La-Roche AG). EBV positivity was determined by the EBV encoding region (EBER) in situ hybridisation (ISH) (ZytoVision Zytofast PLUS CISH Implementation Kit HRP-DAB T-1063–40 and ZytoFast EBV Probe T-1114–400).

### Evaluation of immunohistochemistry

The immunostainings of the tumour samples were scored independently by assessors blinded to the clinical data (JB and JH). In case of differences in the scoring results between assessors, the specific spots were re-evaluated and discussed until reaching consensus. Two to six spots were assessed, and the rounded average was used for analysis. Microsatellite instability (MSI) was assessed by evaluating the expression of MLH1, MSH1, MSH6, and PMS2. We considered the expression positive when more than 5% of tumour cells stained positively compared with both external and internal controls, indicating a mismatch repair proficiency (MMRp). If at least one staining out of four was classified as negative, the sample was classified as mismatch repair deficient (MMRd), which was interpreted as evidence of MSI [15, 16]. E-cadherin staining was scored according to the membranous expression [17] and categorised based on the percentage of tumour cells showing staining in categories as follows: 1) no staining, 2) < 10% of tumour cells with positive membranous staining, 2) 10–50% of tumour cells showing staining, and 4) > 50% of tumour cells positively stained. Ki67 and p53 stainings were scored based on the level of positively stained tumour cells as follows: 1) no staining, 2) < 10% of tumour cells stained, 3) 10–50% of tumour cells stained, and 4) > 50% of tumour cells stained. The p53 staining was interpreted as aberrant when there was either no staining or staining was strong (> 50% of tumour cells stained), and as wild-type when < 50% of tumour cells were stained [18–20]. Based on the EBER*ISH* staining of tumour cells, EBV in situ hybridisation was classified as either positive or negative. To identify tumour cells in TMA spots with morphologically abnormal tissue, we used pan-cytokeratin staining.

### Determining the phenotypic subtypes

**Fig. 1:**
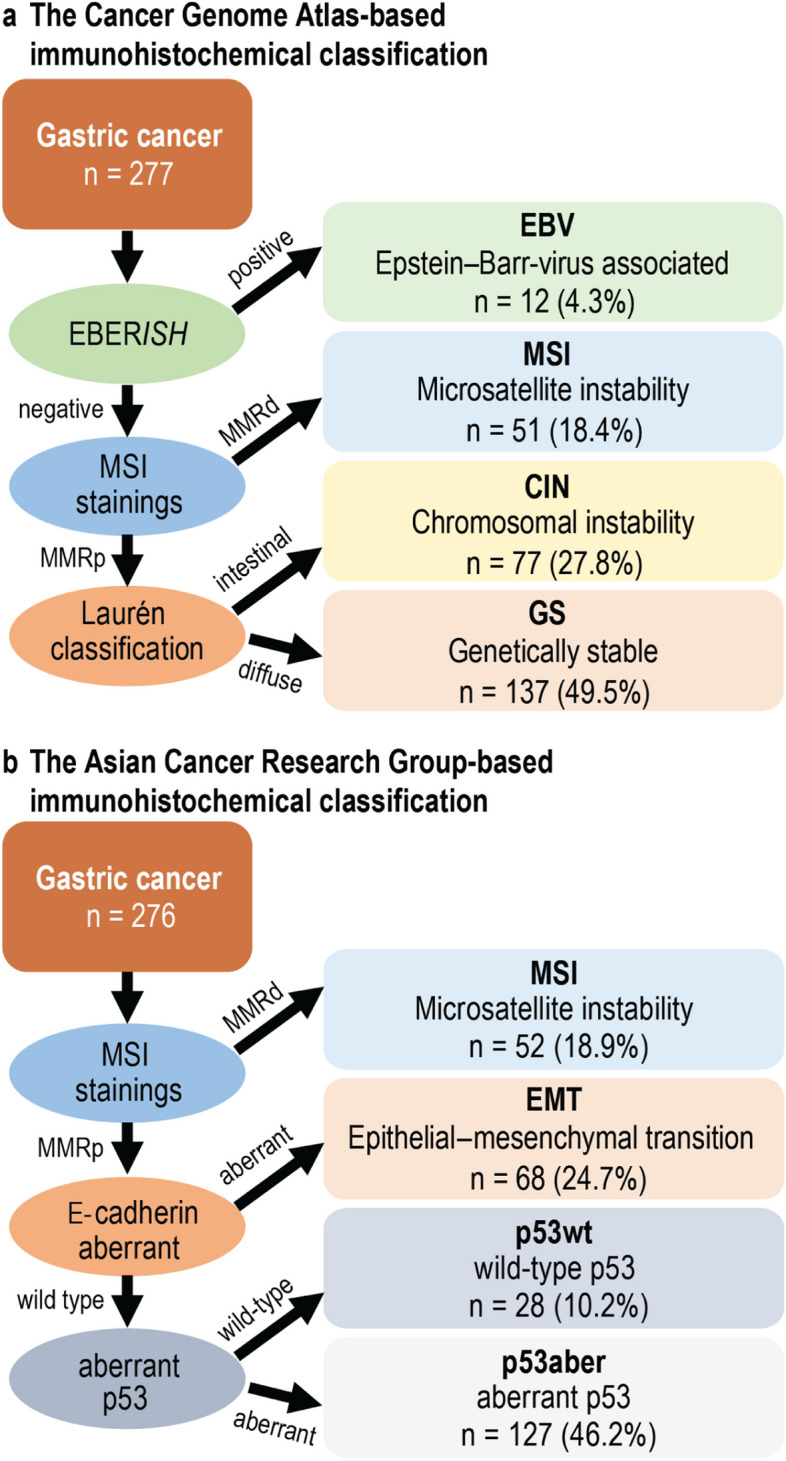
Flowchart of immunohistochemical molecular subtypes. Flowchart presents the subtyping according to **a**) The Cancer Genome Atlas and **b**) Asian Cancer Research Group classifications. Abbreviations: EBER*ISH*, Epstein–Barr virus encoding region in situ hybridisation; EBV, Epstein–Barr virus; MSI, microsatellite instability; MMRd/p, mismatch repair deficient/proficient; CIN, chromosomal instability; GS, genetically stable; EMT, epithelial–mesenchymal transition; p53aber, aberrant p53; p53wt, wild-type p53

In accordance with previous studies, some key elements in the TCGA and ACRG molecular classifications were deployed to form immunohistochemically classified subtypes within our cohort. In the original TCGA molecular subtyping (Fig. 1), EBV-positive patients form the first group. The second group comprises MSI-positive patients, who were interpreted as those patients for whom an abnormal expression appeared in at least one of the four MMR stainings. In the TCGA subtypes, we found that the GS subtype consisted almost exclusively of patients with a diffuse histology and the CIN subtype with an intestinal histology. The third and fourth subtypes, CIN and GS, were determined according to their Laurén classification as intestinal or diffuse and other subtypes. The Laurén classification or a similar classification based on E-cadherin staining was previously used to determine these subtypes [9–12].

Similarly, the ACRG classification defines four subtypes, which in our study were determined using similar immunohistochemical methods as previously reported [9–12]. The first group, the MSI-positive group, was classified similarly to the TCGA-based classification by the negative staining of at least one of the four MMR stainings. The second group is EMT-positive, defined by aberrant E-cadherin staining. The third and fourth groups are defined as wild-type p53 (p53wt) and aberrant p53 (p53aber), respectively, based on the p53 staining.

**Fig. 2:**
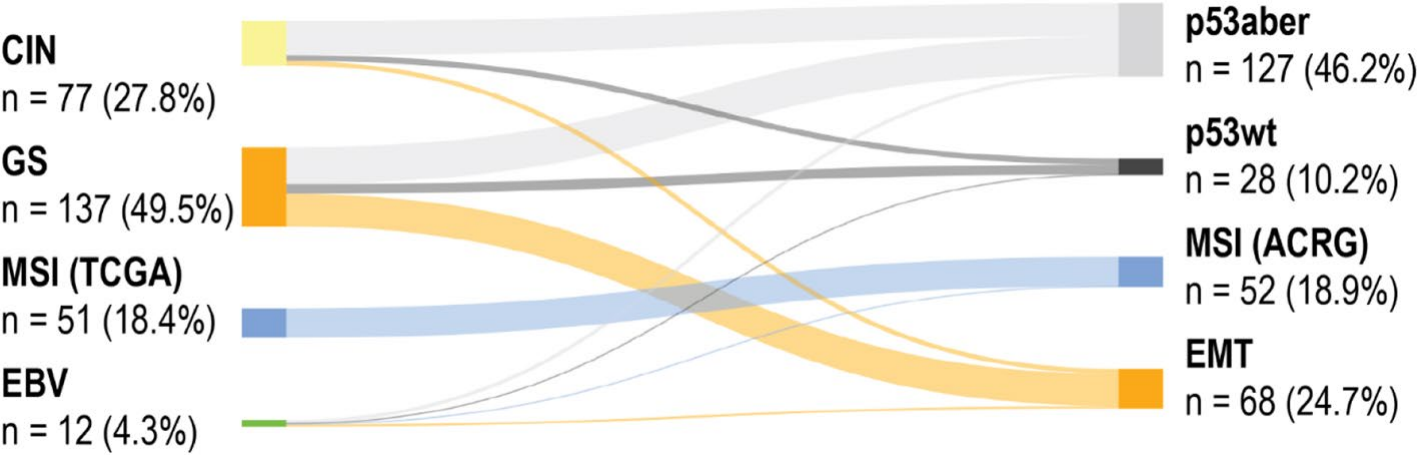
Sankey diagram of connections between subtypes. Width of the connecting line indicates how many patients had different subtypes in the Cancer Genome Atlas and Asian Cancer Research Group–based classifications. Abbreviations: CIN, chromosomal instability; GS, genetically stable; MSI, microsatellite instability; EBV, Epstein–Barr virus; p53aber, aberrant p53; p53wt, wild-type p53; EMT, epithelial–mesenchymal transition

Almost all of the patients in the EMT subtype are also in the GS subtype. Likewise, most patients in the CIN subtype are also in the aberrant p53 subtype. MSI-positive patients form nearly identical groups with just one patient in the EBV-positive subtype. Because both GS and aberrant p53 are the largest subtypes in each classification with around half of the total number of patients, they also considerably overlapped one another (Fig. 2). The phenotypic subtype was successfully determined in 277 patients (97.9%).

### Statistical analysis

We used two-tailed *p* values, considering *p* < 0.05 as statistically significant. Statistical evaluations were calculated using IBM’s statistical software (IBM SPSS Statistics Version 28, International Business Machines Corp., NY, USA). Associations between groups were assessed using the chi-square analysis, the linear-by-linear analysis for ordered parameters such as the cancer stage, the Fisher’s exact test when less than five individuals were included in a single group, or a one-way analysis of variance (ANOVA) for continuous variables. For the univariate and multivariate analyses, we employed the Cox proportional hazard’s regression analysis to determine the disease-specific survival (DSS). We defined DSS as the time from surgery until death from gastric cancer or until the end of the follow-up period. The Cox regression assumption of a constant hazard over time was assessed by plotting the Schoenfeld residuals across time and testing for a trend. No significant deviations from the proportional hazard assumptions were identified. Interactions were considered in the multivariable models, but no statistically significant interactions were detected after applying the Bonferroni correction for multiple testing. For figures with Kaplan–Meier curves, statistical significance was calculated using the log-rank analysis.

## Results

According to our IHC-based TCGA classification, we found 12 patients (4.3%) with the EBV-positive subtype, 51 (18.4%) with the MSI-positive subtype, 77 (27.8%) with CIN or with an intestinal histology, and 137 (49.5%) with a GS or diffuse histology. According to our IHC-based ACRG classification, we found 52 patients (18.9%) with the MSI-positive subtype, 68 (24.7%) with the EMT-positive subtype, 28 (10.2%) with wild-type p53, and 127 (46.2%) with the aberrant p53 subtype (Fig. 1).

### Associations of the Cancer Genome Atlas and Asian Cancer Research Group subtypes with clinicopathological variables

According to our IHC-based TCGA classification, a younger age (< 67 years) was associated with GS, whereas CIN was associated with an older (≥ 67 years) patient age (*p* = 0.002; Table 1). The CIN and EBV subtypes were associated with male sex, while GS was more common among female patients (*p* = 0.021). Stage I cancer associated with CIN patients (*p* = 0.017; Additional file 3) and was less prevalent in the EBV and MSI subtypes. Local pT1 and pT2 tumours associated with CIN (*p* = 0.003; Additional file 3), and locally advanced pT4 tumours associated with the MSI subtype (*p* = 0.012; Additional file 4). All EBV-positive patients presented with lymph node metastases (*p* = 0.019: Additional file 5). In addition, an intestinal histology associated with the EBV subtype (*p* = 0.002).

According to our IHC-based ACRG classification, male sex associated with the aberrant p53 subtype and female sex with the wild-type p53 and EMT subtypes (*p* = 0.043, Table 2). Intestinal histology associated with the aberrant p53 subtype and a diffuse histology with EMT (*p* < 0.001). We noted no association between the ACRG classification and age or stage (Table 2). The MSI subtype associated with a low proliferation index (*p* < 0.001; Additional file 4).

**Table 2:** Associations between ACRG subtypes and clinicopathological variables

	**p53aber**	**p53wt**	**MSI**	**EMT**	*p* value^a^
	*n* = 127 (46.2%)	*n* = 28 (10.2%)	*n* = 52 (18.9%)	*n* = 68 (24.7%)	
**Age**					
< 67	58 (42.3)	17 (12.4)	23 (16.8)	39 (28.5)	0.222
≥ 67	69 (50.0)	11 (8.0)	29 (21.0)	29 (21.0)	
**Sex**					
Male	72 (53.7)	10 (7.5)	26 (19.4)	26 (19.4)	**0.043**
Female	55 (39.0)	18 (12.8)	26 (18.4)	42 (29.8)	
**Stage**					
I	30 (61.2)	6 (12.2)	4 (8.2)	9 (18.4)	0.227
II	31 (48.4)	5 (7.8)	16 (25.0)	12 (18.8)	
III	42 (40.4)	11 (10.6)	19 (18.3)	32 (30.8)	
IV	24 (42.1)	6 (10.5)	12 (21.1)	15 (26.3)	
**Tumour invasion (pT)**					
1	23 (62.2)	5 (13.5)	1 (2.7)	8 (21.6)	0.094
2	20 (48.8)	5 (12.2)	8 (19.5)	8 (19.5)	
3	44 (50.6)	8 (9.2)	17 (19.5)	18 (20.7)	
4	40 (36.4)	10 (9.1)	26 (23.6)	34 (30.9)	
**Lymph node metastases (pN)**					
No	45 (51.7)	9 (10.3)	17 (19.5)	16 (18.4)	0.519
Yes	79 (44.1)	19 (10.6)	34 (19.0)	47 (26.3)	
**Distant metastases (M)**					
No	103 (47.2)	22 (10.1)	40 (18.3)	53 (24.3)	0.916
Yes	24 (42.1)	6 (10.5)	12 (21.1)	15 (26.3)	
**Laurén classification**					
Intestinal	64 (58.2)	11 (10.0)	24 (21.8)	11 (10.0)	** < 0.001**
Diffuse and mixed	63 (38.2)	17 (10.3)	28 (17.0)	57 (34.5)	
**EBV*ish***					
EBV negative	121 (46.0)	27 (10.3)	51 (19.4)	64 (24.3)	0.753
EBV positive	6 (50.0)	1 (8.3)	1 (8.3)	4 (33.3)	
**Ki67 expression**					
< 10%	29 (40.8)	3 (4.2)	19 (26.8)	20 (28.2)	**0.031**
≥ 10%	80 (51.3)	20 (12.8)	27 (17.3)	29 (18.6)	
**p53 expression**					
No staining	94 (56.3)	0 (0.0)	35 (21.0)	38 (22.8)	N/A
< 10%	0 (0.0)	9 (34.6)	5 (19.2)	12 (46.2)	
10–50%	0 (0.0)	19 (52.8)	5 (13.9)	12 (33.3)	
> 50%	33 (71.7)	0 (0.0)	7 (15.2)	6 (13.0)	

*Abbreviations*: *ACRG* Asian Cancer Research Group, *p53aber* aberrant p53, *p53wt* wild-type p53, *MSI* microsatellite instability, *EMT* epithelial–mesenchymal transition, *EBVish* Epstein–Barr virus in situ hybridisation

^a^Pearson's chi-square

The aberrant p53 subtype associated with a lower TNM stage (*p* = 0.027), and local pT1 tumours (*p* = 0.006; Additional file 6). The EMT-positive subtype associated with a diffuse histology (*p* < 0.001) and wild-type p53 (*p* = 0.004; Additional file 7).

### Survival analysis

**Fig. 3:**
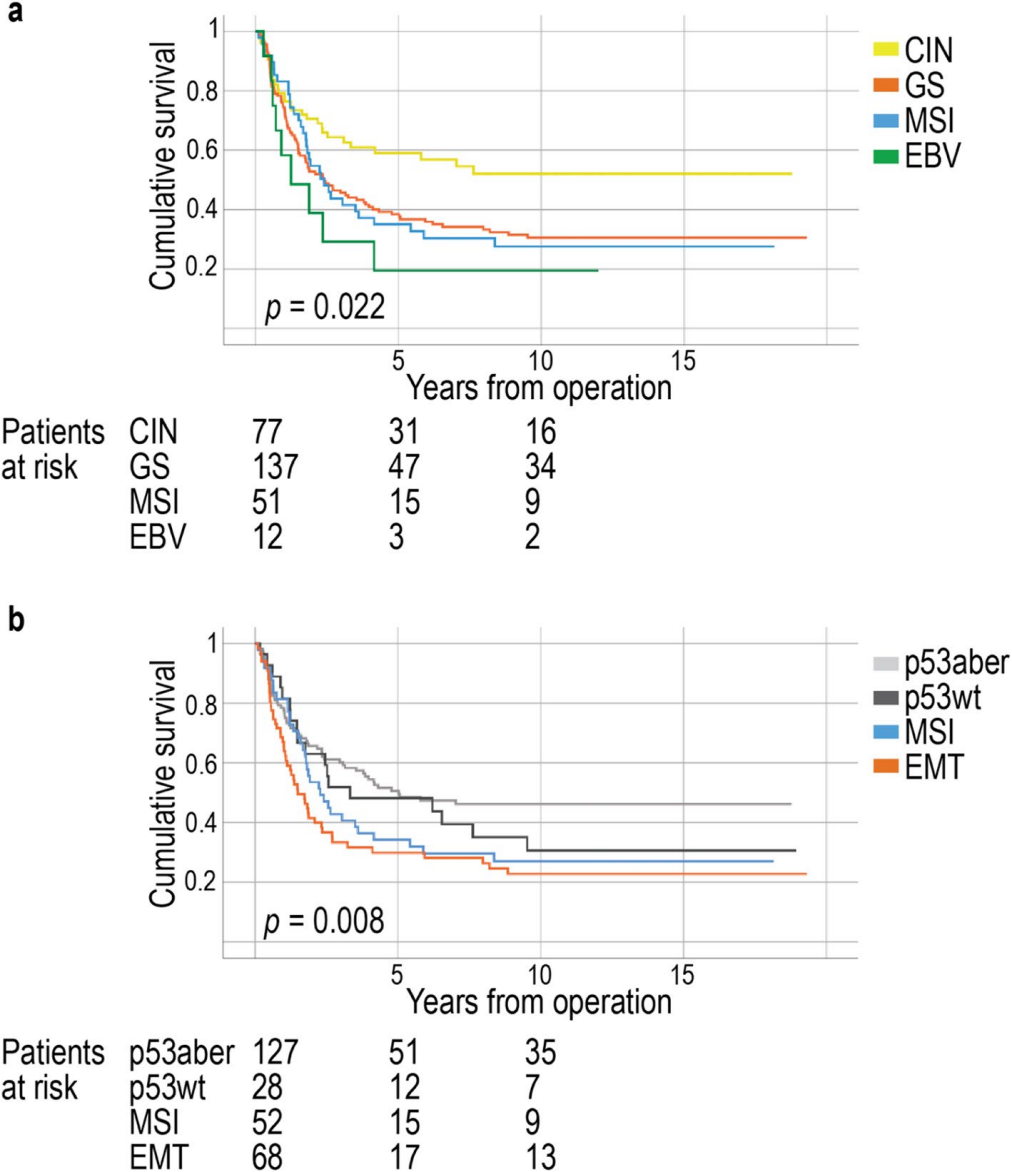
Disease-specific survival of patients according to subtypes. Disease-specific survival of **a**) subtypes based on the Cancer Genome Atlas classification and **b**) subtypes based on the Asian Cancer Research Group classification. Survival curves were drawn according to the Kaplan–Meier method and the *p* values were calculated using the log-rank test. Abbreviations: EBV, Epstein–Barr virus; MSI, microsatellite instability; CIN, chromosomal instability; GS, genetically stable; EMT, epithelial–mesenchymal transition; p53aber, aberrant p53; p53wt, wild-type p53

For the TCGA subtypes, the five-year DSS among patients with the CIN subtype was 59.1% (95% CI 48.4–72.2%), 38.4% (95% CI 30.9–47.8%) for patients with the GS subtype, 35.0% (95% CI 23.6–51.9%) among patients with MSI, and 19.4% (95% CI 5.7–66.4%) for patients with EBV (log-rank test: *p* = 0.022, Fig. 3a). Similar results regarding survival were obtained from the Cox univariate survival analysis (Table 3).

**Table 3:** Univariable Cox regression analysis for disease-specific survival

	**Univariate survival analysis**		
	HR	95% CI	*p* value
**Age**			
< 67	1.00		
≥ 67	1.33	0.99–1.79	0.054
**Stage**			
I	1.00		
II	5.43	2.25–13.1	** < 0.001**
III	15.8	6.87–36.2	** < 0.001**
IV	46.3	19.7–109	** < 0.001**
**Laurén classification**			
Intestinal	1.00		
Diffuse and mixed	1.45	1.06–1.98	**0.020**
**MMR**			
MMRp	1.00		
MMRd	1.18	0.81–1.73	0.383
**EBV*ish***			
EBV negative	1.00		
EBV positive	1.68	0.86–3.29	0.133
**E-cadherin expression**			
No staining	1.00		
< 10%	0.92	0.55–1.54	0.759
10–50%	0.52	0.33–0.84	**0.007**
> 50%	0.57	0.39–0.84	**0.004**
**p53 expression**			
Aberrant	1.00		
Wild-type	1.29	0.91–1.82	0.155
**TCGA molecular subtypes**			
CIN	1.00		
GS	1.73	1.15–2.60	**0.009**
MSI	1.74	1.06–2.84	**0.027**
EBV	2.49	1.19–5.25	**0.016**
**ACRG molecular subtypes**			
p53aber	1.00		
p53wt	1.27	0.75–2.15	0.375
MSI	1.51	0.99–2.30	0.055
EMT	1.90	1.30–2.77	** < 0.001**

*Abbreviations*: *HR* hazard ratio, *CI* confidence interval, *MMR* mismatch repair, *MMRp* mismatch repair proficient, *MMRd* mismatch repair deficient, *EBVish* Epstein–Barr virus in situ hybridisation, *TCGA* The Cancer Genome Atlas, *CIN* chromosomal Instability, *GS* genetically stable, *EBV* Epstein–Barr virus-positive, *ACRG* Asian Cancer Research Group, *p53aber* aberrant p53, *p53wt* wild-type p53, *EMT* epithelial–mesenchymal transition

According to the IHC-based ACRG classification, the five-year DSS among patients with the aberrant p53 subtype was 50.6% (95% CI 42.2–60.8%), 48.2% (95% CI 32.6–71.3%) among those with the wild-type p53 subtype, 34.3% (95% CI 23.1–51.0%) among patients with MSI, and 29.9% (95% CI 20.5–43.8%) among patients with EMT (log-rank test:* p* = 0.008, Fig. 3b). The Cox univariate survival analysis showed similar results related to patient survival in different subtypes (Table 3).

In the univariate survival analysis, patients with a moderate (HR 0.52 [95% CI 0.33–0.84]; *p* = 0.007) or high (HR 0.57 [95% CI 0.39–0.84]; *p* = 0.004) membranous E-cadherin expression exhibited a better survival compared with those with negative staining for membranous E-cadherin (Table 3). By contrast, none of the other variables concerning the MMR status, p53 expression nor EBV positivity individually served as significant markers for survival (Table 3).

The subgroup analysis of the TCGA subtypes revealed that non-metastatic patients with the CIN subtype experienced significantly better survival than all other subtypes, whereas patients with the EBV-positive subtype exhibited a worse survival compared with every other subtype (*p* < 0.001; Additional file 8). Other subgroup-specific analyses revealed no results with distinct clinical relevance.

In the multivariate survival analysis according to TCGA, GS served as an independent prognostic marker with an HR of 1.62 (95% CI 1.06–2.46; *p* = 0.025) when compared with CIN (Table 4). In the multivariate survival analysis according to ACRG, EMT served as an independent prognostic marker with an HR of 1.53 (95% CI 1.03–2.29;* p* = 0.037) when compared with the aberrant p53 subtype.

**Table 4:** Multivariable Cox regression analysis for disease-specific survival

Multivariate survival analysis for TCGA				Multivariate survival analysis for ACRG			
	HR	95% CI	*p* value		HR	95% CI	*p* value
**Age**				**Age**			
< 67	1.00			< 67	1.00		
≥ 67	2.34	1.67–3.27	** < 0.001**	≥ 67	2.46	1.75–3.45	** < 0.001**
**Sex**				**Sex**			
Male	1.00			Male	1.00		
Female	1.34	0.97–1.85	0.077	Female	1.30	0.94–1.80	0.110
**Stage**				**Stage**			
I	1.00			I	1.00		
II	4.50	1.70–11.9	**0.002**	II	4.41	1.67–11.7	**0.003**
III	16.3	6.54–40.6	** < 0.001**	III	16.5	6.62–41.2	** < 0.001**
IV	65.7	25.2–171	** < 0.001**	IV	69.6	26.7–182	** < 0.001**
**TCGA molecular subtypes**				**ACRG molecular subtypes**			
CIN	1.00			p53aber	1.00		
GS	1.62	1.06–2.46	**0.025**	p53wt	1.02	0.60–1.73	0.948
MSI	1.33	0.81–2.19	0.261	MSI	1.15	0.74–1.77	0.534
EBV	1.36	0.62–3.02	0.446	EMT	1.87	1.26–2.76	**0.002**

*Abbreviations*: *HR* hazard ratio, *CI* confidence interval, *TCGA* The Cancer Genome Atlas, *CIN* chromosomal instability, *GS* genetically stable, *MSI* microsatellite instability, *EBV* Epstein–Barr virus-positive, *ACRG* Asian Cancer Research Group, *p53aber* aberrant p53, *p53wt* wild-type p53, *EMT* epithelial–mesenchymal transition

## Discussion

In this study, we found significant prognostic differences between various phenotypically defined molecular subtypes. A better understanding of the clinically significant prognostic biomarkers may help us to better target treatments to different patient subgroups. To achieve this, we proposed a set of specific marker proteins which can be stained using standard immunohistochemistry to easily classify different molecular subtypes. The TCGA and ACRG subtypings are based on genome sequencing and specific mutational profiles, associated with different phenotypical and clinical findings. Previous studies on the molecular subtypes in gastric cancer have been either unsuccessful in identifying significant effects on survival or the results from different studies have conflicted or even contradicted one another [7, 9, 11].

Our results reveal a distribution of patients with different subtypes comparable to previous studies [7–9]. Pretzsch et al. [21] also used IHC to classify patients into subtypes according to the ACRG classification. They, too, used MSI stainings and p53 staining to classify the four ACRG subtypes, but instead of E-cadherin staining they used tumour budding to determine the EMT group. They did not investigate the possible prognostic effect of their classification. In our results, it does not appear that E-cadherin staining (the EMT subtype) yields any additional benefit compared to the Laurén classification (GS subtype).

Zhao et al. [22] used a TMA-based method in a Chinese patient population to identify molecular subtypes according to the ACRG classification. Instead of p53 expression, they examined p21, but otherwise the classification followed the same set-up as ours. They found that the MSI group exhibited the best prognosis, whereas patients in the EMT group exhibited the worst prognosis. They also found an association between the EMT subtype and a more advanced stage of disease resulting in a worse survival. In contrast to our method, p21 expression was divided into high and low expression levels, and not as aberrant or wild-type. The original ACRG subtyping includes the p53 status of patients, with aberrant p53 patients exhibiting the best survival in our patient cohort. The CIN subtype of the TCGA classification is associated with a p53 mutation, which emerged in our dataset as well (*p* = 0.031; Additional file 4).

Wang et al. [11] determined both the TCGA and ACRG subtypes using NGS and IHC methods, respectively. However, they did not compare these two methods against each other in determining the subtypes. Their results concerning survival were aligned with other studies concentrating on Asian populations: patients in the EBV and MSI subtypes exhibited a better survival, whereas patients in EMT and GS exhibited the worst survival. Results concerning patients in the EMT and GS groups exhibiting a worse survival are similar to our results. They identified a clear difference between mutated *TP53* and wild-type patients given that the *TP53* mutation has been associated with a worse survival. However, the difference between immunohistochemically determined molecular subtypes is less clear.

Birkman et al. [9] used a combination of both the ACRG and TCGA classifications to construct their immunohistochemical subtypes. They were not able to show any prognostic effect except that patients with an intestinal Laurén histology and MSI exhibited a better prognosis compared to MSS. They reported finding no difference in survival between wild-type p53 and aberrant tumours. By contrast, our results revealed that patients with the aberrant p53 subtype exhibited the best prognosis*.* Multiple studies have found that p53 IHC staining is a reliable way to determine the *TP53* mutational status in different cancers [23, 24]. However, recent studies reported no association between immunohistochemical findings and genomic alterations [25]. Our results, nonetheless, show a statistically significant difference in survival when using p53 staining in the context of subtypes based on the ACRG classification.

Sun et al. [26] created a combined classification based on TCGA and ACRG subtypes with five subtypes: EBV, MSI, EMT, aberrant p53, and wild-type p53. In their patient cohort of just 165 patients, they had only two EBV-positive patients resulting in the dismissal of this group. Their results concerning survival contradict ours, whereby patients with wild-type p53 had the best prognosis instead of aberrant p53 patients. Pinto et al. [27] also used the same combined classification to distinguish five groups following Sun et al., using NGS and IHC to determine subgroups. Patients’ survival was best in the MSI and EBV subtypes and worst in EMT and wild-type p53 subtypes, mirroring our results. Pinto et al. additionally recommended a schematic to decide possible treatment paths for patients. Ramos et al. [10] also examined IHC subtypes based on both TCGA and ACRG classifications alone and in combination. Their results are well in line with previously described studies showing that patients with MSI had the best survival and patients with EMT/GS had the worst survival. None of the aforementioned studies were able to show any clear superiority between the two sets of subtypes in terms of their prognostic value.

EBV is associated with many cancers and diseases [28]. EBV-positive patients form a small but well-defined subgroup with gastric cancer. At least 90% of people in Western countries have been infected with EBV by early adulthood [29]. Following the primary viral infection, EBV may permanently remain in a latent form in B-cells and possibly the epithelial cells of the digestive and respiratory tracts [30]. The viral genome is inserted as a circular episome in the host cell nucleus or can even be incorporated in the host cell’s genome. There is no way to eradicate EBV from the epithelium, which is possible with *H. pylori* using antibiotics. Furthermore, there is no vaccination against EBV infection. It remains unknown why a latent infection reactivates in some individuals, although it is known to associate with chronic gastritis and may thus promote the development of gastric cancer. In some gastric cancer studies, EBV positivity associates with a stronger immune response, and, thus, with a better prognosis [31–33]. However, our results identified EBV as the subtype with the worst prognosis, with a five-year survival of only 19.4%. Only one patient in the EBV-positive subtype group had distant metastases and almost all patients had an intestinal histology (*p* = 0.002; Additional file 5). Both variables serve as independent markers for a better prognosis, yet EBV exhibits the worst prognosis among all subtypes. A subgroup analysis on patients with non-metastatic disease showed that the EBV subtype had a significantly worse survival compared with all other TCGA subtypes (Additional file 8). Our findings revealed that patients with the EBV subtype exhibit a worse prognosis, a finding previously reported in only one study [34]. A poor prognosis and a known association with a strong immunological response emphasises EBV-positive gastric cancer’s role as a potential target for immuno-oncological treatments.

MSI is widely considered a marker for a good prognosis [7, 8, 10, 11]. In our data, however, we found no association with a better survival. Conversely, our results showed that MMR deficiency associated with locally advanced pT4 cancers, and less commonly with local pT1 cancer. We identified only one MSI-positive patient among the EBV-positive group. Otherwise, MSI-positive patients formed identical groups in both the TCGA- and ACRG-based classifications (Fig. 2). Very few (3.6%) patients received neoadjuvant chemotherapy, currently standard treatment for T2–4 cancers.

In this study, we used the TMA method, where two to six 1-mm-diameter spots were analysed from each individual patient. The spots in the TMA were selected as representative of the entire tumour, allowing for the effective evaluation of different parts of the tumour. This also allowed us to analyse large patient cohorts. Our study comprised a large patient cohort, for which we collected comprehensive clinical data and reliable follow-up information. In our study, we demonstrated that immunohistochemical analysis can be used to identify molecular subtypes of gastric cancer. The method is inexpensive and fast, while revealing significant information for clinical decision-making. The immunohistochemical determination of molecular subtypes is much less costly than methods based on genome sequencing. Regarding clinical implementation, the TCGA classification might be more applicable since one of the determining criteria in our study was the Laurén classification, a standard feature evaluated from every patient.

Current poor survival rates among gastric cancer patients demand better treatment modalities and diagnostics. Ultimately, our findings support using new types of molecular subtyping, which might create a new way of understanding—and treating—gastric cancer on a molecular rather than histological level.

## Conclusions

According to our IHC-based TCGA determination of molecular subtypes, patients with a chromosomal instability exhibited the best prognosis, and, according to our ACRG determination of molecular subtypes, patients with aberrant p53 exhibited the best prognosis. The determination of molecular subtypes based on immunohistochemistry is inexpensive and time-saving. Information on the molecular subtype may help stratify patients for targeted treatments. However, our study does not reveal that either molecular subtyping is better than the other. Thus, further development of the most optimal grouping of different molecular subtypes is still needed.

## Data Availability

The datasets supporting the conclusions of this article are included within the article and its additional files. Other data used in this study is available from the corresponding author upon a reasonable request.

## Abbreviations

ACRG: Asian Cancer Research Group
CI: Confidence interval
CIN: Chromosomal instability
DSS: Disease-specific survival
EBER: Epstein–Barr virus encoding region
EBV: Epstein–Barr virus
EMT: Epithelial–mesenchymal transition
GC: Gastric cancer
GS: Genetically stable
HR: Hazard ratio
IHC: Immunohistochemistry
IQR: Interquartile range
ISH: in situ Hybridisation
MMRd: Mismatch repair deficient
MMRp: Mismatch repair proficient
MSI: Microsatellite instability
NGS: Next generation sequencing
TCGA: The Cancer Genome Atlas
TMA: Tissue microarray
TNM: Tumour-node-metastasis
